# Tissue Type Differences in ABCB1 Expression and Paclitaxel Tissue Pharmacokinetics in Patients With Esophageal Cancer

**DOI:** 10.3389/fphar.2021.759146

**Published:** 2021-11-11

**Authors:** Ruben A. G. van Eerden, Leni van Doorn, Femke M. de Man, Niels Heersche, Michail Doukas, Thierry P. P. van den Bosch, Esther Oomen-de Hoop, Peter de Bruijn, Sander Bins, Eman Ibrahim, Suzan Nikkessen, Lena E. Friberg, Stijn L. W. Koolen, Manon C. W. Spaander, Ron H. J. Mathijssen

**Affiliations:** ^1^ Department of Medical Oncology, Erasmus MC Cancer Institute, Rotterdam, Netherlands; ^2^ Department of Pathology, Erasmus University Medical Center Rotterdam, Rotterdam, Netherlands; ^3^ Department of Pharmacy, Uppsala University, Uppsala, Sweden; ^4^ Department of Gastroenterology and Hepatology Erasmus University Medical Center Rotterdam, Rotterdam, Netherlands; ^5^ Department of Pharmacy, Erasmus University Medical Center Rotterdam, Rotterdam, Netherlands

**Keywords:** tissue pharmacokinetics, intratumoral, paclitaxel, pharmacokinetics, esophageal cancer, ABCB1

## Abstract

**Background:** Data from previous work suggests that there is no correlation between systemic (plasma) paclitaxel exposure and efficacy in patients treated for esophageal cancer. In this trial, we investigated ATP-binding cassette efflux transporter expression and intratumoral pharmacokinetics of paclitaxel to identify changes which could be a first sign of chemoresistance.

**Methods:** Patients with esophageal cancer treated with paclitaxel and carboplatin (± concomitant radiotherapy) were included. During the first and last cycle of weekly paclitaxel, blood samples and biopsies of esophageal mucosa and tumor tissue were taken. Changes in paclitaxel exposure and expression of ABCB1 (P-glycoprotein) over time were studied in both tumor tissue and normal appearing esophageal mucosa.

**Results:** ABCB1 was significantly higher expressed in tumor tissue compared to esophageal tissue, during both the first and last cycle of paclitaxel (cycle 1: *p* < 0.01; cycle 5/6: *p* = 0.01). Interestingly, ABCB1 expression was significantly higher in adenocarcinoma than in squamous cell carcinoma (*p* < 0.01). During the first cycle, a trend towards a higher intratumoral paclitaxel concentration was observed compared to the esophageal mucosa concentration (RD:43%; 95%CI: −3% to 111% *p* = 0.07). Intratumoral and plasma paclitaxel concentrations were significantly correlated during the first cycle (AUC_0–48 h_: *r* = 0.72; *p* < 0.01).

**Conclusion:** Higher ABCB1 expression in tumor tissue, and differences between histological tumor types might partly explain why tumors respond differently to systemic treatment. Resistance by altered intratumoral paclitaxel concentrations could not be demonstrated because the majority of the biopsies taken at the last cycle of paclitaxel did contain a low amount of tumor cells or no tumor.

## Introduction

Esophageal cancer is the 7th most common cause of cancer-related mortality worldwide (Global Burden of Disease Cancer et al., 2018). Paclitaxel in combination with carboplatin and radiotherapy is highly effective in the curative setting of esophageal cancer, and in combination with carboplatin alone it has shown moderate efficacy both during induction chemotherapy and in the palliative setting of this tumor type (Polee et al., 2004; van Hagen et al., 2012; Shapiro et al., 2015; de Man et al., 2019). Nonetheless, a substantial part of the patients with esophageal cancer do not benefit from this treatment or show progression of disease short after their treatment has stopped (Chirieac et al., 2005; de Man et al., 2019; Toxopeus et al., 2019).

Paclitaxel acts by the inhibition of cell proliferation, by promoting the stabilization of cellular microtubules and the concentration-dependent induction of multipolar spindles which eventually leads to apoptosis (Jordan and Wilson, 2004; Weaver, 2014; Zasadil et al., 2014). Paclitaxel is also known for its induction of drug resistance (Barbuti and Chen, 2015), although the exact mechanisms are unknown. Major factors probably causing paclitaxel resistance are alterations in stability of the microtubule network, reduced function of apoptotic proteins (e.g., B-cell leukemia/lymphoma 2 (Bcl-2), cellular tumor antigen (p53)), and overexpression of transmembrane efflux-pumps of the ATP-binding cassette (ABC) subfamily (Gottesman et al., 2002; Huisman et al., 2005; Barbuti and Chen, 2015).

ABC-efflux transporters are essential in the protection of the cell against xenobiotics (Schinkel and Jonker, 2003). ABCB1 (P-glycoprotein) is one of the subtypes in the ABC-efflux transporter family (Schinkel and Jonker, 2003). ABCB1 is expressed in the plasma membrane of human cells and is known for its diversity in substrates that can be transported via this efflux transporter (Schinkel and Jonker, 2003). Overexpression of ABCB1 contributes to chemotherapy resistance of cancer cells *in vitro* and was related to worse survival of cancer patients in several studies (Trock et al., 1997; Schinkel and Jonker, 2003; Schaich et al., 2005; Haber et al., 2006; Stordal et al., 2012; Barbuti and Chen, 2015). *In vivo* studies demonstrated that inhibition or induction of ABCB1 in multidrug resistant tumor cells influences the intratumoral paclitaxel exposure (Huisman et al., 2005; Tiwari et al., 2013). Nevertheless, intratumoral pharmacokinetics of chemotherapeutical agents, and the relation between intratumoral chemotherapy exposure and ABC efflux transporter activity remains largely unknown, especially in the clinical setting.

In contrast to tissue pharmacokinetics, the systemic pharmacokinetics of paclitaxel are well known and characterized by a large inter-individual variability (Henningsson et al., 2003; de Graan et al., 2013). Moreover, commonly seen hematological toxicity and peripheral neuropathy have been linked with the time above a specific paclitaxel plasma concentration (i.e., >0.05 µM) (Gianni et al., 1995; Mielke et al., 2005). To determine the best dose for an individual patient it is often suggested to tailor the dose of paclitaxel based on the systemic pharmacokinetic exposure. This strategy improved the risk-benefit profile of non-small cell lung cancer patients treated with paclitaxel (Joerger et al., 2016). However, this is probably only a surrogate for the intratumoral exposure (Mathijssen et al., 2011). Additionally, in a previous study no correlation between systemic paclitaxel clearance and esophageal cancer response was shown (Toxopeus et al., 2019).

Currently, knowledge about the intratumoral concentrations of paclitaxel, the influence of intratumoral paclitaxel concentration on the effectiveness of the treatment and the correlation between ABC efflux transporters and intratumoral paclitaxel is lacking. Therefore, there is an urgent need to investigate and elucidate the intratumoral paclitaxel pharmacokinetics.

In this exploratory study we assessed both ABC efflux transporter expression, and intratumoral and esophageal mucosa paclitaxel concentrations over time, to identify changes in paclitaxel concentrations and/or differences between tissue types which could potentially be a sign of the development of drug resistance in esophageal carcinoma.

## Methods

We performed a single center pharmacokinetic study in patients diagnosed with esophageal cancer for whom treatment with weekly paclitaxel and carboplatin was indicated. The study was performed between October 2017 and September 2019 at the Erasmus MC Cancer Institute, Rotterdam, Netherlands. The Medical Ethics Committee and the board of directors of the Erasmus MC approved the study protocol. The study was performed in accordance with the International Conference on Harmonization Good Clinical Practice guidelines, the Declaration of Helsinki, and all applicable regulations. The trial is registered at the Dutch Trial Registry (www.trialregister.nl number NL5990). All patients provided written informed consent before any study related procedure was pursued.

### Patients

Patients, 18 years or older, were eligible if they were diagnosed with a histologically proven malignancy of the esophagus that was safely accessible by upper endoscopy. They were treated with weekly paclitaxel and carboplatin with or without concomitant radiotherapy in a standard regimen (Supplementary Methods 1) (Polee et al., 2004; van Hagen et al., 2012; de Man et al., 2019). Patients had to have an Eastern Cooperative Oncology Group (ECOG) performance status of 0 or 1. Patients were excluded if the tumor caused esophageal stenosis prohibiting upper endoscopy, if they previously received radiotherapy on the esophagus, if they had a history of bleeding diathesis, or if they used medication or supplements which could interact with paclitaxel during the study period.

### Study Design

The primary objective of the study was to demonstrate a 25% reduction of the intratumoral concentration of paclitaxel in the last cycle of weekly paclitaxel compared to the first cycle of paclitaxel in esophageal cancer patients. Secondary objectives of our study were to: 1. compare intratumoral paclitaxel concentrations with paclitaxel concentrations in normal appearing esophageal mucosa, 2. compare paclitaxel concentrations in non-tumoral mucosa per study cycle, 3. correlate intratumoral concentrations of paclitaxel with systemic paclitaxel pharmacokinetics per study cycle, 4. to investigate ABCB1 expression over time, 5. compare ABCB1 expression between tumor tissue and non-tumoral esophageal mucosa tissue, and 6. compare ABCB1 expression between different histological types of esophageal cancer.

All included patients were seen at the outpatient clinic prior to each chemotherapy cycle. During cycle 1 and the last cycle (i.e., cycle 5 or 6), patients were admitted to the hospital to perform blood withdrawals for pharmacokinetic purposes and to undergo an upper endoscopy to obtain biopsies of the tumor and normal appearing esophageal mucosa for pharmacokinetic purposes and pathological assessments. Patients were evaluable for the primary endpoint if the biopsies were successfully obtained during the first and the last cycle of their weekly paclitaxel treatment.

### Biopsy Procedure

Upper endoscopy was planned at 4 h after the start of paclitaxel administration. Sedation with midazolam and fentanyl was allowed during the endoscopy procedure. During the procedure, a total of 2–4 biopsies of the tumor --with a mean diameter of 6 mm-- were taken by an experienced and dedicated gastroenterologist. Biopsies of normal appearing esophageal mucosa (visual inspection by the gastroenterologist) were taken at least 5 cm proximal or distally from the visible tumor area. These biopsies were of the same size and same numbers as the tumor biopsies. Half of the biopsies were directly frozen in liquid nitrogen and stored at < −70°C for pharmacokinetic analysis. The other half of the biopsies were formalin-fixed for pathological assessment. If the gastroenterologist could not identify a macroscopic tumor during the last treatment cycle, samples were taken at the same location as during the first cycle.

### Pharmacokinetic Analysis

Plasma samples were taken before start of the paclitaxel infusion, 30 min after start of administration, 5 min prior to the end of infusion, and 1.5 and 3 h after the end of the administration of paclitaxel. The timing of blood sampling as well as tissue sampling were comparable when the anti-allergic infusion regimen was used. Blood samples were collected in 4 ml lithium heparin tubes and plasma was collected after centrifugation at 2,500*g (4°C) for 10 min and stored at < −70°C until analysis. Paclitaxel concentrations were measured using a validated liquid chromatography-mass spectrometry method (Sparreboom et al., 1998). Systemic exposure was expressed as area under the curve from pre-infusion to 48 h (AUC_0–48h_) and estimated using a previously developed population PK model developed in NONMEM (Henningsson et al., 2003). The analysis took the anti-allergic infusion regimen into account.

Tissue biopsies were homogenized in 400 µL of blank human plasma with a tissue-lyser (Qiagen, Germany) and a stainless-steel bead (5 mm) for 90 s at 60 Hz. Homogenized tissue samples were further processed as plasma samples as described above.

### Pathological Analysis

To determine the expression of ABCB1 an automated immunostainer (the Ventana Benchmark ULTRA, Ventana Medical Systems Inc., Arizona, United States) was used. Sequential 4 µm thick (FFPE) sections were stained for ABCB1 using Optiview universal DAB detection Kit (#760–700, Ventana).

In brief, following deparaffinization and heat-induced antigen retrieval with CC1 (#950–500, Ventana) for 32 min, the tissue samples were incubated with the ABCB1 antibody (Company: Novusbio; Type: anti mouse; Clone: OTI1A7; Lot number: W001; Dilution: 1/9,600) for another 32 min at 37°C. Incubation was followed by hematoxylin II counter stain for 8 min and then a blue coloring reagent for 8 min according to the manufactures instructions (Ventana). Positive controls were used on every slide.

After immunohistochemical staining the percentage of positive stained cells of interest and the intensity of the staining per biopsy were evaluated (by R.A.G.v.E. and M.D.). The biopsies were scored according to the immunoreactive score (IRS) described by Remmele and Stegner (Remmele and Stegner, 1987).

### Statistical Analysis

This study was powered to detect a 25% decrease of the intratumoral concentrations of paclitaxel in the last treatment cycle compared to the first treatment cycle. Since we had no information on forehand on the variability of the intratumoral paclitaxel concentrations, we assumed an intrapatient standard deviation of 30% in intratumoral paclitaxel concentrations. Given a power of 80% and two-sided significance level of 5%, at least 14 evaluable patients were required for the primary objective.

Log-transformation was used for data regarding tissue (tumor and normal appearing esophagus mucosa tissue) paclitaxel concentrations and AUC_0–48h_, since we assumed that these data followed a lognormal distribution. A paired *t*-test was used to compare tissue paclitaxel concentrations, and systemic exposure (i.e., AUC_0–48h_) for the total study population. Mean differences with corresponding 95% confidence intervals (CI) were exponentiated to calculate the geometric mean ratio with 95% CI for these ratios. Geometric mean (GEM) ratios represent relative differences (RD) as a percentage. Comparisons between the first cycle and last cycle were made for intratumoral concentrations, healthy esophageal mucosa tissue concentrations, and plasma AUC_0–48h_ using paired *t*-test. The intratumoral paclitaxel concentration was also compared with normal appearing esophageal tissue concentration during cycle 1 and the last cycle using the same test. The intratumoral concentrations observed in adenocarcinoma and squamous cell carcinoma were compared to each other using an independent *t*-test. To compare the ABC efflux transporter expression between the types of tissues and the cycles of chemotherapy the Wilcoxon signed-rank test was used. The correlation between systemic pharmacokinetics and tissue paclitaxel concentrations was estimated using Pearson’s correlation coefficients. The correlation between immunohistochemical expression and intratumoral paclitaxel concentrations was estimated using Spearman’s correlation coefficient given the ordinal immunohistochemical data used for this analysis.

The datasets used and/or analyzed during the current study are available from the corresponding author on reasonable request.

## Results

### Patient Characteristics

In total 15 patients were included, of whom 14 patients were evaluable. One patient withdrew informed consent after the first cycle of chemotherapy and gastroscopy within the study. Table 1 displays all baseline characteristics.

**TABLE 1:** Baseline characteristics.

Characteristics (*n* = 14)	No. (%)
**Gender**	
Male	13 (93%)
Female	1 (7%)
**Age (years)**	
Median [IQR]	70 [64 – 76]
**BMI (kg/m** ^**2**^ **)**	
Median [IQR]	27.7 [25.3 – 33.8]
**BSA (m** ^**2**^ **)**	
Median [IQR]	2.1 [1.85 – 2.20]
**Tumor location**	
Mid esophageal tumor^a^	3 (21%)
Distal esophageal tumor^b^	11 (79%)
**Histological subtype**	
Adenocarcinoma	9 (64%)
Squamous cell carcinoma	5 (36%)
**Treatment regimen**	
CTx	3 (21%)
dCRT	2 (14%)
nCRT	9 (64%)

a Mid esophageal tumor is defined as tumor located at 24–32 cm from the teeth row.

b Distal esophageal tumor is located between 32 and 40 cm form the teeth row.

Abbreviations: BMI, body mass index; BSA, body surface area; CTx, chemotherapy; dCRT, definitive chemoradiotherapy; IQR, interquartile range; nCRT, neoadjuvant chemoradiotherapy; No., number of cases.

The tumors were predominantly located in the distal esophagus (79%). Nine out of the 14 patients (67%) were diagnosed with an adenocarcinoma, while the remaining patients were diagnosed with a squamous cell carcinoma of the esophagus. The majority of the included patients were male (93%) and were treated with paclitaxel (50 mg/m^2^), carboplatin (AUC2) and concomitant radiotherapy (78%).

### Tissue Biopsies

The time between start of infusion and biopsies was comparable between cycle 1 (median 4.8 h; IQR 4.3–5.1 h) and the last cycle (median 4.3 h; IQR 3.7–4.8 h). A summary of the location of the analyzed biopsies and pathological assessments is presented in Supplementary Table S1. The amount of tissue obtained during the biopsy procedure differed between normal esophageal mucosa and tumor tissue, and between cycles (cycle 1 tumor tissue: median 6.4 mg (IQR: 4.3–7.9 mg); cycle 1 esophageal mucosa: median 2.9 mg (IQR: 2.5–4.3 mg); last cycle tumor tissue: median 5.6 mg (IQR: 2.0–7.1 mg); last cycle esophageal mucosa: median 2.0 mg (IQR: 0.99–2.3 mg)). All biopsies of the tumor at the first cycle contained cancer cells (median cancer cell percentage 60%; IQR 30–85%). Biopsies of normally appearing esophageal mucosa during cycle 1 nonetheless contained tumor cells in two patients: subject 4 (20% tumor cells) and subject 15 (30% tumor cells). Of the tumor biopsies taken at the last treatment cycle, only the biopsies of six patients (43%) contained tumor cells, which is probably a positive result of the treatment. Of these six biopsies containing tumor cells, 5 samples contained maximum 10% tumor cells and one sample contained 80% tumor cells. The esophageal mucosa samples taken at the last cycle were all tumor cell negative, except one which contained 1% tumor cells. Necrosis was present in a minority of the biopsies, i.e., in 5 tumor samples and 1 normal mucosal sample during cycle 1 and in 4 tumor samples and 3 normal mucosal samples during the last cycle. In patients treated with concomitant radiotherapy, tumor samples showed limited necrosis percentages but instead showed active inflammation or ulceration.

### Tissue Pharmacokinetics

Paclitaxel could be measured in all biopsy samples. One sample (esophageal mucosa cycle 5; subject 12) was excluded from all analyses due to a low amount of tissue (i.e., 0.04 mg) resulting in an unreliable quantification of the paclitaxel concentration. No statistical analyses were performed involving the tumor samples taken during the last cycle given the low amount of tumor cells observed in these biopsies. During the first cycle, a trend towards a higher intratumoral paclitaxel concentration was seen compared to the esophageal mucosa paclitaxel concentration (RD: 43.44%; 95% CI: −2.60–111.22%; *p* = 0.07; Table 2) (excluding Barrett’s esophagus biopsies; RD: 58%; 95% CI: 3–145%; *p* = 0.04). The GEM paclitaxel concentration in normal esophageal mucosa during the first cycle was 2.03 ng/mg (95% CI: 1.38–2.98 ng/mg) while the intratumoral GEM paclitaxel concentration was 2.91 ng/mg (95% CI: 2.22–3.83 ng/mg). The intratumoral paclitaxel concentration in adenocarcinoma samples was not significantly different from the concentrations measured in squamous cell carcinoma samples during the first cycle (RD: -11%; 95% CI: −53–70%; *p* = 0.70; Table 2). The paclitaxel concentration in esophageal mucosa during the last cycle of chemotherapy (GEM: 1.89 ng/mg (95% CI: 1.26–2.85 ng/mg)) was not significantly different from the concentration measured during the first cycle in esophageal mucosa (RD: −10%; 95% CI: −47–53%; *p* = 0.68; Table 2).

**TABLE 2:** Comparisons of tissue pharmacokinetics.

	Relative difference	95% confidence interval	*p*-value
Esophageal PTX last cycle vs. esophageal PTX first cycle	−10%	−47% to 53%	0.68
Tumoral PTX first cycle vs. esophageal PTX first cycle	43%	−3% to 111%	0.07
Adenocarcinoma PTX first cycle vs squamous cell carcinoma PTX first cycle	−11%	−53% to 70%	0.70

PTX, paclitaxel

### Immunohistochemical Staining

**FIGURE 1:**
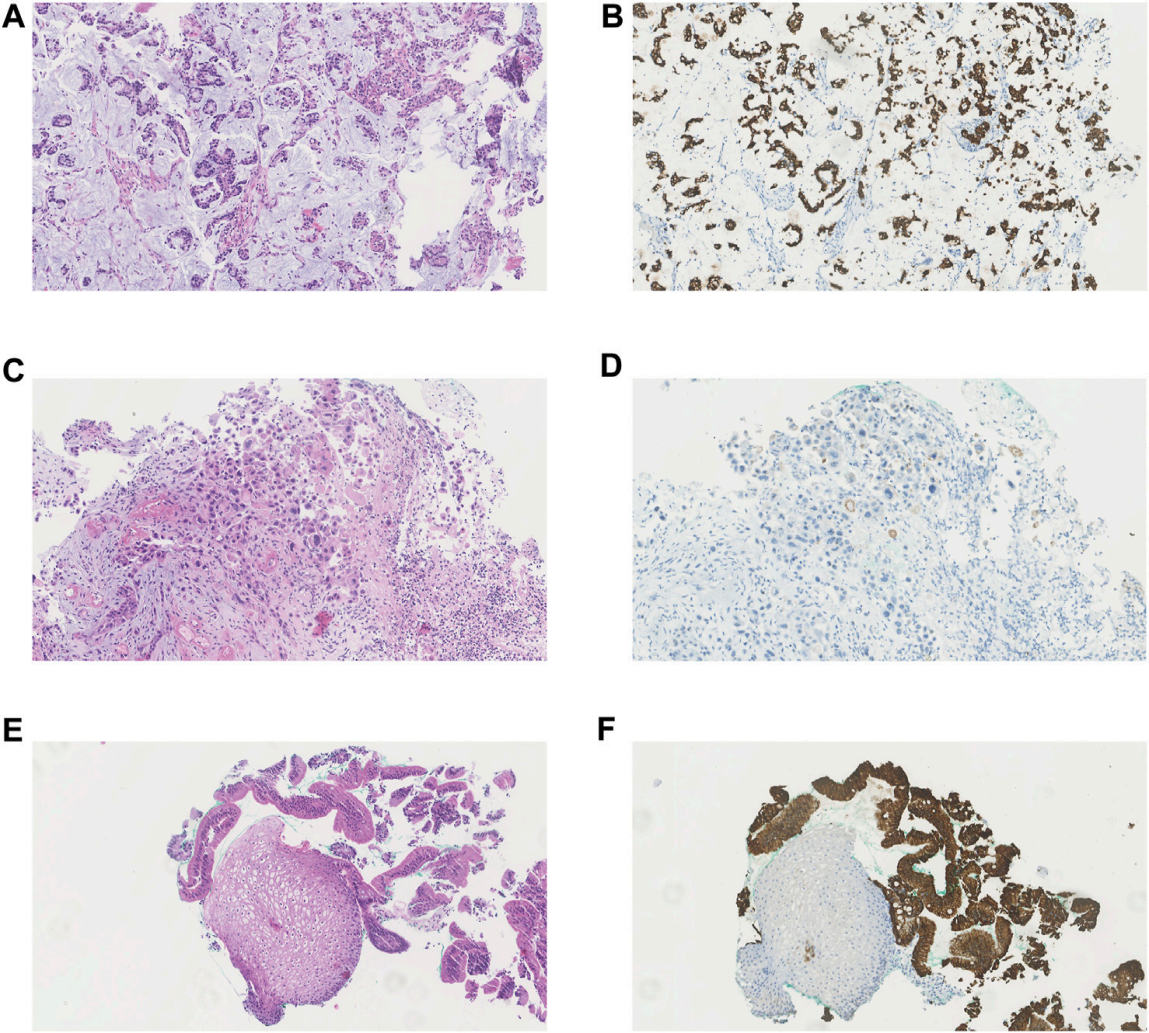
Haematoxylin and eosin (H&E) staining and immunohistochemical staining of ABCB1 in different types of investigated tissue. **(A)** H&E staining of adenocarcinoma **(B)** ABCB1 immunohistochemical staining of adenocarcinoma **(C)** H&E staining of squamous cell carcinoma **(D)** ABCB1 immunohistochemical staining of squamous cell carcinoma **(E)** H&E staining of healthy esophageal mucosa and Barrett esophagus **(F)** ABCB1 immunohistochemical staining of healthy esophageal mucosa and Barrett esophagus.

A summary of all immunohistochemical scores per biopsy is presented in Table 3. Figure 1 depicts the H&E staining and the ABCB1 staining of a general representable biopsy of an adenocarcinoma (Figures 1A,B), squamous cell carcinoma (Figures 1C,D), healthy esophageal mucosa tissue (Figures 1E,F) and Barrett’s esophagus (Figures 1E,F). During the first cycle, ABCB1 expression in esophageal tumors was significantly higher than in normal esophagus mucosa biopsies (*p* < 0.01). The majority of the normal esophageal tissue biopsies did not express ABCB1 (11 out of 13 (85%)) according to the IRS score during this cycle. The other two esophageal tissue biopsies expressed ABCB1 mildly (*n* = 1) and strongly (*n* = 1 (8%)), respectively, of which the latter biopsy was taken from a Barrett’s esophagus (Figure 1F). ABCB1 staining of tumor samples was strongly positive in all 9 adenocarcinoma samples taken during cycle 1, whereas ABCB1 was expressed significantly less in the squamous cell carcinoma samples (*p* < 0.01), i.e., moderately (*n* = 2), weakly (*n* = 1) or not at all (*n* = 1).

**TABLE 3:** Immunohistochemical score of ABCB1 per biopsy.

Subject	Cycle	Tumor				Esophagus			
		Percentage positive cells	Score positive cells	Intensity score	IRS score	Percentage positive cells	Score positive cells	Intensity score	IRS score
1	Last cycle	no tumor	0	no tumor	NA	0%	0	0	0
2	First cycle	100%	4	3	12	0%	0	0	0
3	First cycle	40%	2	3	6	0%	0	0	0
3	Last cycle	40%	2	3	6	0%	0	0	0
4	First cycle	no tumor	0	no tumor	NA	0%	0	0	0
4	Last cycle	no tumor	0	no tumor	NA	0%	0	0	0
5	First cycle	5%	1	3	3	1%	1	3	3
5	Last cycle	no tumor	0	no tumor	NA	0%	0	0	0
6	First cycle	100%	4	3	12	0%	0	0	0
6	Last cycle	100%	4	3	12	1%	1	3	3
7	First cycle	60%	3	3	9	0%	0	0	0
7	Last cycle	100%	4	3	12	0%	0	0	0
8	First cycle	5%	1	1	1	0%	0	0	0
8	Last cycle	no tumor	0	no tumor	NA	0%	0	0	0
9	First cycle	100%	4	3	12	no tissue	no tissue	no tissue	NA
9	Last cycle	100%	4	3	12	0%	0	0	0
10	First cycle	100%	4	3	12	0%	0	0	0
10	Last cycle	100%	4	3	12	50%	2	2	4
11	First cycle	100%	4	3	12	0%	0	0	0
11	Last cycle	100%	4	3	12	0%	0	0	0
12	First cycle	100%	4	3	12	0%	0	0	0
12	Last cycle	100%	4	3	12	0%	0	0	0
13	First cycle	20%	2	2	4	0%	0	0	0
13	Last cycle	no tumor	0	no tumor	NA	no tissue	no tissue	no tissue	NA
14	First cycle	100%	4	3	12	0%	0	0	0
14	Last cycle	100%	4	3	12	10%	2	3	6
15	First cycle	100%	4	3	12	100%	4	3	12
15	Last cycle	100%	4	3	12	100%	4	3	12

The IRS (immunoreactive score) indicates different categories of ABCB1 expression (i.e., IRS 0–1 = negative for ABCB1; IRS 2–3 = mild ABCB1 expression; IRS 4–8 = moderate ABCB1 expression; IRS 9–12 = strong ABCB1 expression).

Nine tumor samples were evaluable for immunohistochemistry at the last cycle: the 8 evaluable adenocarcinoma samples all remained strongly positive for ABCB1. The single evaluable squamous cell carcinoma sample expressed ABCB1 moderately which also corresponds with the ABCB1 expression observed during the first cycle of chemotherapy (Table 3). From the esophageal tissue biopsies taken after the last cycle, 9 biopsies (69%) were negative for ABCB1, while 1 sample (8%) was mildly positive, 2 samples (16%) moderately positive and 1 (Barrett’s esophagus) sample (i.e., biopsy subject 15) (8%) was strongly positive for ABCB1 expression.

In line with the results seen during the first cycle, the expression of ABCB1 during the last cycle was also significantly higher in tumor samples compared to healthy esophageal tissue (*p* = 0.01).

### Plasma Pharmacokinetics

The geometric mean AUC_0–48h_ of paclitaxel was 2,898 ng·h/mL (95% CI: 2,171–3,868 ng·h/mL) during the first cycle, and was similar (2,946 ng·h/mL (95% CI: 2,186–3,969 ng·h/mL)) during the last cycle (RD: 1.66%; 95%CI: −5.41–9.25%; *p* = 0.631).

### Correlation Between Tissue- and Plasma Pharmacokinetics

**FIGURE 2:**
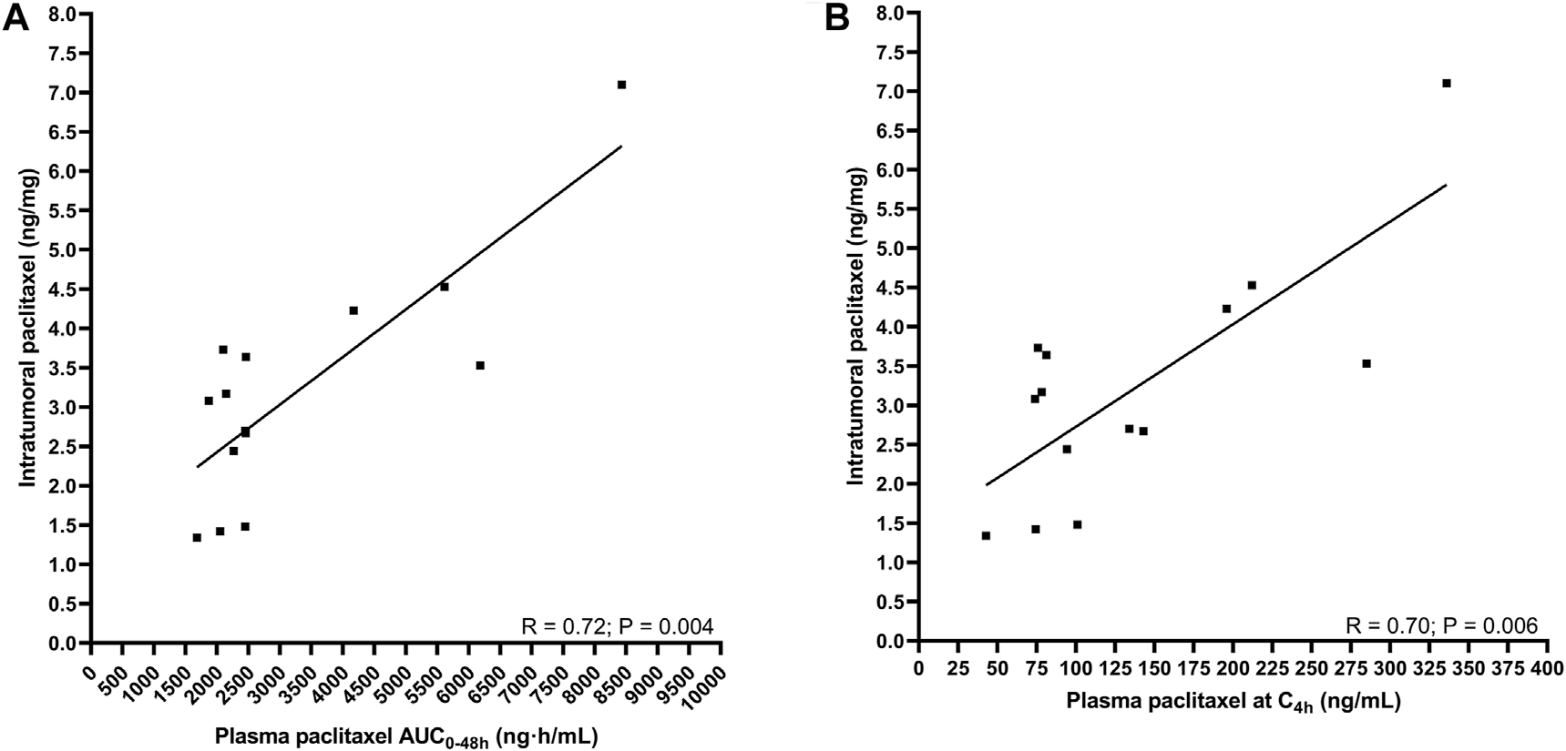
The correlation between intratumoral pharmacokinetics and plasma pharmacokinetics of paclitaxel. **(A)** intratumoral paclitaxel concentration and AUC_0–48 h_ **(B)** intratumoral paclitaxel concentration and concentration at 4 h after start infusion.

No correlation could be determined between plasma pharmacokinetics (i.e., AUC_0–48h_) or the plasma concentration measured around the biopsy procedure (C_4h_)) and the paclitaxel concentration in esophageal mucosa during the first and last cycle of paclitaxel. Interestingly, the intratumoral paclitaxel concentration was strongly positively correlated to the plasma pharmacokinetics (AUC_0–48* *h_: R = 0.72; *p* < 0.01 and C_4h_: R = 0.70; *p* < 0.01) during cycle 1 (Figures 2A,B).

## Discussion

In this study, we demonstrated that ABCB1 efflux transporter expression is significantly higher in adenocarcinoma of the esophagus compared to squamous cell carcinoma of the esophagus. Moreover, the expression of ABCB1 by esophageal carcinomas is higher compared to normal-appearing esophageal mucosa. We could not demonstrate an alteration of intratumoral paclitaxel as first sign of resistance due to the low tumor cell percentage in the second biopsies. Nevertheless, we may have (partly) explained the effectivity of this taxane in esophageal cancer since the paclitaxel concentration in non-tumoral esophageal mucosa is lower than in tumor tissue, and a strong correlation between plasma pharmacokinetics and intratumoral paclitaxel concentration was seen.

We have tried to identify pharmacokinetic mechanisms of resistance to paclitaxel in esophageal cancer. A major factor contributing to the occurrence of paclitaxel resistance in solid tumors is overexpression of ABC efflux transporters, which could potentially lower the intratumoral drug concentration (Gottesman et al., 2002; Huisman et al., 2005; Barbuti and Chen, 2015). Previous studies have reported expression of ABCB1 in adenocarcinoma of the esophagus, as well as in squamous cell carcinoma, while no expression of ABCB1 was described in esophageal mucosa (Atlas; Vrana et al., 2018). In line with these results, we have demonstrated that ABCB1 expression was higher in esophageal carcinoma than in normal esophageal mucosa. However, we have also demonstrated a significantly higher expression of ABCB1 in adenocarcinoma than in squamous cell carcinoma of the esophagus. Interestingly, in the CROSS trial a significantly higher complete response rate in patients with squamous cell carcinoma of the esophagus than in those with esophageal adenocarcinoma was found (van Hagen et al., 2012). Further, in the long-term data of the CROSS trial also a clinically relevant difference (adenocarcinoma: median overall survival of 43 months versus squamous cell carcinoma: 82 months median overall survival) between the two histological types seems to exist (Shapiro et al., 2015). Therefore, it could be speculated that ABCB1 expression might have contributed to the differences in complete response rate and median survival between the two histological types. Several other studies investigating different regimens of repeated preoperative chemotherapy and radiotherapy in esophageal carcinoma could not identify a survival difference between those two histological subtypes (Cooper et al., 1999; Reynolds et al., 2007; Xi et al., 2017). This difference could possibly be explained by the fact that those studies administered cisplatin and fluoropyrimidines as chemotherapeutical agents which are both not substrates of ABCB1 (Marzolini et al., 2004).

The difference in ABCB1 expression between the different types of tissues could also be used to improve treatment with carboplatin and paclitaxel in esophageal cancer. A higher ABCB1 tissue expression is expected to result in a lower tissue drug concentration that could lead to a lower efficacy of the drug. Several studies demonstrated that in cell lines overexpressing ABC efflux transporters, inhibition of ABC transporters results in higher intratumoral paclitaxel exposure (Stordal et al., 2012; Tiwari et al., 2013). Increasing the intratumoral paclitaxel exposure by inhibition of ABCB1 expression might enhance the efficacy of the treatment, and thereby reducing a substantial part of patients who do not benefit from the treatment with paclitaxel and carboplatin. Since normal esophageal mucosa does not express ABCB1, it is not expected that inhibition of this ABC efflux transporter results in an increased chemotherapeutical exposure in the healthy esophageal mucosa (EMBL-EBI Expression Atlas, 2021). Nevertheless, previous research demonstrated that the use of MDRT (multidrug resistance transporters) inhibitors are complicated by several factors (Choi and Yu, 2014). The first generation of these inhibitors are characterized by the high doses needed with only limited efficacy, the severe toxicity profile of those compounds and the pharmacokinetic effects on other drugs (Choi and Yu, 2014). Since these drugs affect drug transporters they have an (potentially negative) effect on the absorption, distribution, metabolism and elimination of others drugs used in patients (Choi and Yu, 2014). Furthermore, it is always important to realize that these transporters are also expressed at other sites than tumors. ABCB1 transporters are also expressed by liver tissue and kidney tissue which could increase paclitaxel related toxicity in those organs which is undesirable given their essential function (EMBL-EBI Expression Atlas, 2021). Newer generations of MDRT inhibitors are characterized by milder toxicity profiles and reduced effects on the overall pharmacokinetics properties and therefore also the pharmacokinetics of other drugs (Choi and Yu, 2014). Nonetheless, the efficacy of these newer generation of MDRT inhibitors remained also limited which might be caused by heterogeneity of the tumor cells regarding ABCB1 expression, drug penetration, and other simultaneous existing resistance mechanisms (Choi and Yu, 2014).

Contrary to the aforementioned expected influence of ABCB1 expression on tissue paclitaxel exposure, the intratumoral paclitaxel concentration is higher than the paclitaxel concentration in esophageal mucosa despite the higher ABCB1 expression in tumor tissue. One of the factors that might explain the discrepancy between the expectations and the observed results could be tumor vessel permeability. The permeability of vessels in the tumor is higher compared to healthy esophageal tissue that could make it more easily for paclitaxel to distribute into the tumor tissue (Pasqualini et al., 2002). The fact that we identified a strong correlation between systemic paclitaxel pharmacokinetics and intratumoral pharmacokinetics could also point to a high vessel permeability in the tumor. Moreover, in line with our findings, it was previously demonstrated that the intratumoral cisplatin concentration in tumor tissue of patients diagnosed with esophagus carcinoma and treated with cisplatin and 5-fluorouracil (5-FU) was higher compared to the concentration in healthy esophagus tissue (Troger et al., 1991). Increased permeability of tumor tissue may also be induced by fractionated radiotherapy (Debbage et al., 2000; Ng et al., 2007). In line with the described effects of radiotherapy, the intratumoral doxorubicin distribution was improved by radiotherapy (Potiron et al., 2019).

Alterations over time in ABCB1 expression or intratumoral paclitaxel concentrations might also be a first sign of resistance of the tumor. Nonetheless, we could not identify an alteration in ABCB1 expression over time. This may be the result of the relatively short treatment period in our study. In addition, we used a low chemotherapy dose. In contrast, Di Nicolantonio et al. did observe a significant increase in mRNA levels of ABCB1 in paired samples of adenocarcinoma of the esophagus after chemotherapy in an *in vitro* experiment and therefore may not be concordant with our clinical study results (Di Nicolantonio et al., 2005). Moreover, Langer et al. also reported no alterations in ABCB1 expression after neoadjuvant chemotherapy in their clinical study (Langer et al., 2007).

A comparison between the intratumoral paclitaxel concentration during the first cycle and last cycle was hampered by a low amount of tumor cells observed in tumor biopsies taken during the last cycle. Previous studies demonstrated that up to 28% of the patients who undergo chemoradiotherapy have a complete pathological response after completion of their treatment (van Hagen et al., 2012; Shapiro et al., 2014). Therefore, it is most likely that the low amount of tumor cells observed in the biopsies taken during the last cycle is an effect of the chemoradiotherapy treatment. Due to this low tumor cell percentage in the tumor biopsies it could be doubted if the paclitaxel concentrations measured represents the intratumoral paclitaxel concentration. Given that biopsies are homogenized before paclitaxel quantification, it is likely that the paclitaxel concentration measured represents the concentration inside the most dominant type of tissue, which is probably non tumorous tissue in the intended tumor biopsies of the last cycle. Therefore, we could not investigate the alteration in intratumoral paclitaxel concentrations over time which could be a sign of chemotherapy resistance.

Previously, it has also been attempted to investigate the intratumoral paclitaxel pharmacokinetics via several mathematical models which predict the distribution of the drug inside the tumor (Popilski and Stepensky, 2015). However, these models have limited accuracy probably due to simplification of the multiple factors involved in intratumoral drug distribution and can therefore not replace tumor biopsies for intratumoral pharmacokinetic analysis (Popilski and Stepensky, 2015). However, bioanalytical methods should be further improved so that even if a low amount of tumor tissue has been obtained, the intratumoral paclitaxel could be accurately measured without the influence of paclitaxel in the surrounding tissue on the measured intratumoral paclitaxel concentration. Matrix-assisted laser desorption/ionization (MALDI) mass spectrometry might be a tool to achieve such an analytical improvement.

In conclusion, we found a significantly higher ABCB1 expression in esophageal adenocarcinomas than in squamous cell carcinomas, which might be causally related to a better treatment effectivity of paclitaxel in the latter. Resistance by reduced intratumoral paclitaxel concentrations could not be demonstrated because of the low tumor percentage at the last cycle of paclitaxel. Further research investigating the ABCB1 expression in esophageal carcinoma and esophageal mucosa tissue is warranted to elucidate the relationship between response and ABCB1 status.

## Data Availability

The datasets generated during and/or analysed during the current study are available from the corresponding author on reasonable request.
