# Supplementary material for: Tissue Type Differences in ABCB1 Expression and Paclitaxel Tissue Pharmacokinetics in Patients With Esophageal Cancer
Source: Front Pharmacol. 2021 Nov 11;12:759146. doi: 10.3389/fphar.2021.759146 (PMC8632367; doi:10.3389/fphar.2021.759146)
Supplement: Supplementary file 1 [file DataSheet1.DOCX]

**Supplementary Methods 1:**

**Treatment:**

To be eligible for this study patients could be treated with different schedules of chemotherapy. Choice for either of these schedules was made by the treating physician based on indication. The following schedules of paclitaxel were used in the study, depending on indication:

- Weekly paclitaxel 50 mg/m^2^ in a 1-hour infusion was given together with carboplatin area under the curve (AUC) of 2 mg/mL/min concomitant with a total 3D conformal radiation dose of 41.4 Gy or 50.4 Gy given in 23 fractions or 28 fractions of 1.8 Gy each, with 5 fractions administered per week for 5 (in the neoadjuvant setting) or 6 (as definitive chemoradiotherapy) consecutive weeks depending on the indication.(Shapiro et al., 2015)
- Weekly paclitaxel 100 mg/m^2^ in a 1-hour infusion, in combination with carboplatin targeting an AUC of 4 mg/mL/min, for 6 consecutive weeks.(Polee et al., 2004)

If patients experienced an allergic reaction during paclitaxel infusion, paclitaxel was given in a 1.75-hour infusion during the remaining cycles according to local anti-allergic regimen, consisting of:

- 0- 15 min Paclitaxel infusion at 15 mL/hour
- 15- 30 min 50 mL NaCl 0.9% + 2 mg clemastine
- 30-45 min Paclitaxel infusion at 84 mL/hour
- 45-105 min If no allergic reaction follows continue infusion of paclitaxel at 500 mL/hour

| **Subj.** | **Regimen** | **Tissue type** | **Cycle** | **Location PK^α^ (cm)** | **Weight (mg)** | **Location PA^β^ (cm)** | **Tumor cells (%)** | **Necrosis (%)** | **Cycle** | **Location PK^α^ (cm)** | **Weight (mg)** | **Location PA^β^ (cm)** | **Tumor cells (%)** | **Necrosis (%)** |
| --- | --- | --- | --- | --- | --- | --- | --- | --- | --- | --- | --- | --- | --- | --- |
|  |  |  |  |  |  |  |  |  |  |  |  |  |  |  |
| 1 | CTx | Tumor | 1 | 34 | 6.69 | - | - | - | 5 | 35 | 0.97 | 35 | 0 | 100 |
| 1 | CTx | Esophageal mucosa | 1 | 26 | 4.99 | - | - | - | 5 | 28 | 5.34 | 28 | 0 | 0 |
| 3 | CTx | Tumor | 1 | 38 | 9.66 | 36 | 80 | 20 | 6 | 39 | 15.2 | 39 | 0 | - |
| 3 | CTx | Esophageal mucosa | 1 | 30 | 2.94 | 29 | 0 | 0 | 6 | 30 | 1.97 | 30 | - | - |
| 4 | dCRT | Tumor | 1 | 32 | 7.34 | 32 | 10 | 10 | 6 | 28 | 1.46 | 28 | 0 | 10 |
| 4 | dCRT | Esophageal mucosa | 1 | 27 | 4.3 | 27 | 30 | 20 | 6 | 26 | 1.15 | 27 | 0 | 50 |
| 5 | nCRT | Tumor | 1 | 32 | 7.75 | 30 | 70 | 0 | 5 | 30 | 6.9 | 30 | 0 | 0 |
| 5 | nCRT | Esophageal mucosa | 1 | 23 | 0.57 | 24 | 0 | 0 | 5 | 25 | 0.33 | 25 | - | - |
| 6 | CTx | Tumor | 1 | 37 | 4.07 | 35 | 5 | 0 | 6 | 38 | 1.25 | 39 | 80 | 10 |
| 6 | CTx | Esophageal mucosa | 1 | 25 | 3.79 | 25 | 0 | 0 | 6 | 28 | 2.24 | 28 | 1 | 10 |
| 7 | nCRT | Tumor | 1 | 34 | 4.22 | 35 | 50 | 0 | 5 | 33 | 6.42 | 33 | 5 | 0 |
| 7 | nCRT | Esophageal mucosa | 1 | 28 | 2.92 | 28 | 0 | 0 | 5 | 28 | 2.52 | 28 | 0 | 0 |
| 8 | nCRT | Tumor | 1 | 37 | 6.18 | 37 | 90 | 10 | 5 | 37 | 7.11 | 37 | 0 | 0 |
| 8 | nCRT | Esophageal mucosa | 1 | 30 | 4.33 | 30 | 0 | 0 | 5 | 30 | 1.93 | 30 | 0 | 0 |
| 9 | nCRT | Tumor | 1 | 31 | 4.2 | 30 | 60 | 0 | 5 | 30 | 4.83 | 30 | 1 | 0 |
| 9 | nCRT | Esophageal mucosa | 1 | 28 | 2.35 | 25 | 0 | 0 | 5 | 23 | 2.52 | 23 | 0 | 0 |
| 10 | nCRT | Tumor | 1 | 35 | 7.67 | 35 | 100 | 0 | 5 | 39 | 7.7 | 39 | 10 | 0 |
| 10 | nCRT | Esophageal mucosa | 1 | 30 | 5.27 | 29 | 0 | 0 | 5 | 35 | 1.98 | 35 | 0 | 0 |
| 11 | nCRT | Tumor | 1 | 37 | 8.44 | 37 | 70 | 30 | 5 | 37 | 3.88 | 37 | 5 | 0 |
| 11 | nCRT | Esophageal mucosa | 1 | 32 | 1.14 | 32 | - | - | 5 | 32 | 0.56 | 32 | - | 0 |
| 12 | nCRT | Tumor | 1 | 34 | 4.35 | 34/35 | 100 | 0 | 5 | 39 | 6.52 | 35 | 10 | 0 |
| 12 | nCRT | Esophageal mucosa | 1 | 29 | 2.53 | 30 | 0 | 0 | 5 | 30 | 0.04 | 30 | - | - |
| 13 | dCRT | Tumor | 1 | 19 | 9.1 | 19 | 30 | 60 | 5 | 22 | 2.17 | 22 | 0 | - |
| 13 | dCRT | Esophageal mucosa | 1 | 32 | 2.83 | 32 | - | - | 5 | 27 | 1.13 | 27 | 0 | - |
| 14 | nCRT | Tumor | 1 | 38 | 4.81 | 38 | 60 | 0 | 5 | 37 | 7.24 | 37 | 0 | 0 |
| 14 | nCRT | Esophageal mucosa | 1 | 33 | 2.8 | 33 | 0 | 0 | 5 | 33 | 1.27 | 33 | 0 | 50 |
| 15 | nCRT | Tumor | 1 | 26 | 4.52 | 26 | 30 | 0 | 5 | 26 | 3.28 | 26 | 0 | 0 |
| 15 | nCRT | Esophageal mucosa | 1 | 20 | 2.72 | 20 | 20 | 0 | 5 | 20 | 2.11 | 20 | 0 | 0 |

**^α^** = Pharmacokinetic (PK) biopsy, **^β^** = Pathological (PA) biopsy. Abbreviations: CTx = Chemotherapy, dCRT = Definitive chemoradiotherapy, nCRT = Neoadjuvant chemoradiotherapy.

**Supplementary Table 1. Pharmacokinetic tissue sample characteristics**

**Supplementary References:**

Polee, M.B., Sparreboom, A., Eskens, F.A., Hoekstra, R., van de Schaaf, J., Verweij, J., et al. (2004). A phase I and pharmacokinetic study of weekly paclitaxel and carboplatin in patients with metastatic esophageal cancer. *Clin Cancer Res* 10(6)**,** 1928-1934.

Shapiro, J., van Lanschot, J.J.B., Hulshof, M., van Hagen, P., van Berge Henegouwen, M.I., Wijnhoven, B.P.L., et al. (2015). Neoadjuvant chemoradiotherapy plus surgery versus surgery alone for oesophageal or junctional cancer (CROSS): long-term results of a randomised controlled trial. *Lancet Oncol* 16(9)**,** 1090-1098.
